# Assessment of the nursing skill mix in Mozambique using a task analysis methodology

**DOI:** 10.1186/1478-4491-12-5

**Published:** 2014-01-25

**Authors:** Martinho Dgedge, Angel Mendoza, Edgar Necochea, Debora Bossemeyer, Maharifa Rajabo, Judith Fullerton

**Affiliations:** 1 National Directorate of Human Resources, Ministry of Health, Mozambique, Av. Eduardo Mondlane, No. 1008, 2nd Floor, Maputo, Mozambique; 2 Jhpiego Mozambique, Rua Jose Mateus 27, Maputo, Mozambique; 3 Jhpiego/Johns Hopkins University, 1615 Thames Street, Baltimore, MD 21231, USA; 4 Independent Consultant, 7717 Canyon Point Lane, San Diego, CA 92126, USA

## Abstract

**Background:**

The density of the nursing and maternal child health nursing workforce in Mozambique (0.32/1000) is well below the WHO minimum standard of 1 nurse per 1000. Two levels of education were being offered for both nurses and maternal child health nurses, in programmes ranging from 18 to 30 months in length. The health care workforce in Mozambique also includes Medical Technicians and Medical Agents, who are also educated at either basic or mid-level. The Ministry of Health determined the need to document the tasks that each of the six cadres was performing within various health facilities to identify gaps, and duplications, in order to identify strategies for streamlining workforce production, while retaining highest educational and competency standards. The methodology of task analysis (TA) was used to achieve this objective. This article provides information about the TA methodology, and selected outcomes of the very broad study.

**Methods:**

A cross-sectional descriptive task analysis survey was conducted over a 15 month period (2008–2009). A stratified sample of 1295 individuals was recruited from every type of health facility in all of Mozambique’s 10 provinces and in Maputo City. Respondents indicated how frequently they performed any of 233 patient care tasks. Data analysis focused on identifying areas where identical tasks were performed by the various cadres. Analyses addressed frequency of performance, grouped by level of educational preparation, within various types of health facilities.

**Results:**

Task sharing ranged from 74% to 88% between basic and general nurse cadres and from 54% to 88% between maternal and child health nurse cadres, within various health facility types. Conversely, there was distinction between scope of practice for nursing and maternal/child health nursing cadres.

**Conclusion:**

The educational pathways to general nursing and maternal/child health nursing careers were consolidated into one 24 month programme for each career. The scopes of practice were affirmed based on task analysis survey data.

## Background

Mozambique, a country of approximately 21 million people, has undergone substantial developmental growth and change since achieving independence in 1975. The country has seen very strong economic growth in recent years [1], although the country is presently ranked 185 (of 187) on the Human Development Index [2].

The Government has made a commitment for health care reform and improvement in all health and human service sectors, in order to make an impact on the health and wellbeing of its citizens and to reach its goal of providing sufficient and high-quality services to its people, and to achieve the Millennium Development Goals (MDG) [3] by 2015. The Government’s commitment offers great promise of success, as evidenced by recent improvements in various health indicators, including reductions in maternal [4], neonatal and child mortality [5], a 96% immunization coverage rate, a reduction of the malaria mortality rate, expansion of access to tuberculosis (TB) detection and treatment and a significant increase in the number of persons benefiting from antiretroviral treatment [6].

Nevertheless substantial challenges remain. A major barrier that must be addressed is the deficit of human resources in health, in both absolute numbers and in the proportion of providers available to provide specific services [7-10]. The Ministry of Health (MOH) has developed its National Plan for Health Human Resources Development (NPHHRD) through the year 2015 [11], which sets forth an agenda for increasing the total number of health workers, including nurses and other health services personnel.

Recent estimates from the World Health Organization (WHO) for the nursing workforce in Mozambique indicate a total of 6183 personnel (3954 nurses; 2229 midwives) [6,12]. This equals a density of 0.21 nurses per 1000 population (0.33 including midwives), or approximately 20 nurses per 100 000 population. These figures compare to a density of 1.172 nurses per 1000 population for other African countries [6] and a WHO minimum standard of 1 nurse per 1000 [13].

In response to this significant deficit of nursing personnel in the country, the MOH NPHHRD established an ambitious target to double the number of nurses by 2015. In addition to the great need to increase the number of nurses, the MOH was concerned about improving the quality of nursing staff. This is in line with studies suggesting an association between the higher number and more qualified nursing staff with better outcomes of patient care in hospitals [14-16]. The MOH believes that it is necessary to have nursing staff with the knowledge, skills, and leadership to achieve a significant improvement of health services.

The MOH has responsibility for the education of the country’s nurses, maternal/child health nurses and various other cadres of health personnel. At the time the present study was initiated there were two educational pathways for nurses and two for maternal and child health nurses. Nursing training programmes for the basic level were 18 months in length (basic nurse, BN) and were offered at all 13 training institutions in the country to candidates with 10th grade-level of education. A 30-month general nurse (GN) programme (mid-level) was offered in eight of these training institutions. Basic maternal/child health nurse (MCHN-B) training was offered at all 13 training institutions, and required a 20-month period of study. MCHN training at mid-level (MCHN-G) was offered at seven institutions, and was also 30 months in length.

A second cadre of health practitioner also provides client care services in the country, that is, medical agents, basic level (MA-B) and medical technician, mid-level (MT-M). The education requirement for both cadres includes a minimum age of 17 years, and completion of the 10th grade of education. The length of the training programme for medical agents is three semesters of studies (1 ½ years) and five semesters (2 ½ years) for mid-level practitioners.

The MA and MT cadres were included in this study because of the MOH’s interest in obtaining similar information about the six cadres and specifically observing whether there were relevant duplications of tasks between the six scopes of practice. This information was important to define and/or reaffirm the scope of work of the nursing careers. However, the findings presented in this present report focus primarily on the four cadres of nurses that comprised the then-current Mozambique nurse workforce. It was predicated that an efficient and simplified educational process was needed in order to expand the numbers and the quality of nursing staff. The existence of many different careers and levels was perceived to be a hindrance to efficient production. Therefore, the MOH determined to explore all possibilities to achieve synergies and economies in educational programming. The MOH initiated a curriculum revision and update process for the nursing and maternal and child health nursing programmes of study. In preparation for that revision it was considered important to conduct a detailed task analysis (TA) to identify the health care tasks that different careers and levels of nursing and medical agents and technician personnel perform in order to examine gaps and duplications that required correction. This article presents the methodology of TA, using selected findings from the Mozambique study as an example of the utility of this research strategy.

## Methods

Two parallel TA approaches were used in the overarching study. A review of the curricula of studies for the cadres (assessment of knowledge) was conducted to identify the expected outcomes of education. That component of the study is briefly described, but the findings are not reported in the current article.

A task analysis survey (assessment of skills, and the focus of this article) was intended to identify those tasks presently expected to be performed by the various health cadres in the variety of health service settings in the country. This assessment allowed a review of the scopes of practice of the six cadres, in light of the country’s burden of disease and the population’s need of services.

The TA process is widely used in the health professions as a tool to define the scope of practice for various cadres of health workers [17]. The TA methodology is a systematic qualitative and quantitative process to generate information about activities or processes (tasks) that must be implemented to achieve an objective [18,19]. A task is defined as an action that produces a result to achieve an objective, and it has an identifiable beginning and end, thus constitutes a measurable component of responsibilities of a particular job. These responsibilities typically include cognitive (knowledge), manual (skills), and affective domains (attitudes and values) [20].

### Human subjects

Administrative approval to conduct this situation analysis was received from the MOH. The assessment protocol was reviewed and approved as a programme-based evaluation by the Human Subjects review committees of the MOH, Johns Hopkins University and of the technical collaborative agency, the US Centers for Disease Control and Prevention.

### Assessment design

The assessment included both qualitative and quantitative methods. A knowledge consensus workshop was conducted, at which ratings were generated about the importance of including any specific area of content into the curriculum for various cadres. A field survey based on TA methodology generated ratings about the frequency with which the health provider actually used a particular skill. The cross-sectional study was conducted in progressive steps over a 15-month period between January 2008 and March 2009.

### Sampling procedure and sample size

The participants in the national survey on skills were nurses, maternal and child health nurses, medical agents and technicians. The sample included representatives randomly and proportionately selected from among the various categories of health workers from every province, and from every type of healthcare facility, in both urban and rural areas. The interviewers solicited the participation of individuals at their place of work, approaching potential participants according to the stratified sample that had been prepared. Voluntary consent was obtained. The interviews were arranged in a place that accommodated both privacy and comfort, and at a time convenient to the respondent. Interviews were interrupted in the event that the provider was needed for service, and resumed when convenient, so that the full interview was eventually conducted.

### Instruments

Two English/Portuguese dual-language instruments were developed for the purpose of the overarching study. A knowledge assessment tool addressed the fundamental core of knowledge that might need to be included in nursing, maternal and child health nursing or medical agent/technician curricula. Personal interviews with knowledgeable informants and a review of documents generated information that was used in the development of the study instruments, specifically, the core body of knowledge and the set of practical skills that should be the expected outcomes of education for any of the cadres included in the study. This process was implemented for each level (basic and mid-level) of the three cadres (nursing, maternal and child health nursing, and medical technicians).

The content items for the knowledge tool were drawn from policy documents and scope of practice statements for the various cadres of health worker [21], from core curricula of the various education programmes, and from relevant documents, such as job descriptions and the national plan for health care facilities. The content items were modified and expanded through the work of 48 content experts who verified the content validity of the items. The final instrument contained 300 independent items organized into 31 separate groups of related content (that is, *the need to know*). Importance ratings (1 = not important; 2 = somewhat important; 3 = very important) were assigned to each specific knowledge item to indicate its relationship to the fundamental core of information that any one of the six cadres of health workers would need to acquire in order to provide effective and high quality health-care services. Nursing content experts from the expert group completed the rating task for the nurse professions, assigning a value to the importance of including each item of knowledge within the curriculum of each of the four cadres of nursing personnel, using the dual-language version of the instrument described below.

The task tool was comprised of a list of cognitive and practical skills, that is, the practical competencies that these health workers should be expected to acquire. It was generated by extracting information from the same country-specific policy and curriculum documents, and augmented after review of various nursing skills lists promulgated by nursing organizations and other health policy bodies [22-24]. The skills survey tool represented a list of practical activities (tasks) that any of the six cadres of health workers might be expected to perform on the job, (that is, *the need to do).* The skills list contained 233 items, organized into nine separate domains of clinical and community-focused care. A 5-point frequency scale provided ratings about how often any task was performed in a single day (never; at least once a day; at least once a week; at least once a month; or seldom). The classification “never” had four subcategories to provide information on why the health worker did not personally carry out the specific task (was not trained to perform the task; the task is not performed in the index health facility; another provider performed the task; or essential materials were not available). An importance scale intended to measure the relationship between the task and client health outcomes was developed, but large amounts of missing data rendered these findings unreliable.

A single (qualitative) interview question, “*Is there anything else that you are required to do on the job for which you were not prepared during your education program?”,* was added to the task tool. This question was posed to identify skills that should be considered for inclusion in the basic education programme of the various nursing cadres, given that they are tasks required in the workplace in order to provide high-quality client care.

### Instrument translation

The instruments were developed in the English language; then translated into Portuguese by native language speakers, using a cross-cultural forward translation methodology [25,26].

### Field survey

A national field survey was conducted over a 6-week period between November and December 2008. A total of 55 individuals served as field interviewers and field supervisors in their province of work or residence (4 to 6 per province). Two national supervisors provided oversight of the survey process and consultation for field supervisors.

The survey teams participated in a 5-day theoretical and practical skills training workshop, focused on the data collection methods and tools. The training included practice of the technique of conducting a facilitated interview and fieldwork for pilot testing of the usability and clarity of the study instrument. Inter-rater reliability (IRR) in use of the tool was computed for 16 interviews obtained during a one-day field practice at two hospitals in Maputo City and Province. The IRR was computed as agreement of at least 80%, which represents an acceptable minimum standard [27]. The majority of agreements exceeded 90% over the 233-item instrument. Internal reliability of the skills instrument was assessed following the field test, and exceeded Chronbach’s alpha *r* = 0.70 for each of the nine subscales.

### Data management and analysis

Electronic databases were custom designed for this project. SPSS Version 16 (SPSS Inc., Chicago, IL, USA) was used for both data entry and analysis purposes. Data quality control reviews were conducted weekly by a supervisor who was not involved in direct data entry. A 10% sample of items was selected at random from among all data entered during the previous week and double-entered, to assure accuracy and integrity of the data.

Statistical measures included computation of the average rating assigned to each knowledge item, and the proportion of responses to each task rating to determine the frequency with which any task was performed by a member of any cadre in any facility setting. A cross-comparison of the relative ranking of the frequency with which tasks were performed by various cadres was conducted to determine task sharing between and among various cadres. Sub-analyses were conducted to identify variance in the frequency of task performance as a function of facility type (for example, central hospital versus rural health post) or location (urban versus rural).

Specific sub-analyses were performed to determine the degree to which tasks were shared between the two cadres of nurses and the two cadres of maternal/child health nurses, as well as among the four cadres in general. The intention of these sub-analyses was to identify the degree of skill-mix that was inherent in the existing educational curricula [28,29].

## Results

### Characteristics of the sample

A total of 1,295 individuals provided data for the assessment (BN = 431; GN = 192; MCHN-B = 275; MCHN-G = 118; MA-B = 177; MT-M = 102). The acceptance rate was greater than 99% of all individuals who were approached for participation.

Respondents ranged in age from 19 to 70 years. The majority of nurses and MCH nurses were female (634/1016; 62.4%); three quarters of medical agents and technicians were male (n = 216/279; 74.4%). There was no statistically significant difference in the mean age among respondents, by province. The years of professional work experience on the unit to which the respondent was assigned at the time of interview ranged from less than six months (n = 153; 11.8%) to greater than 15 years (n = 68; 5.3%). The majority of respondents had from one to five years of experience on their units (n = 587; 45.4%).

### Task sharing

The findings presented in this article are a very limited example of the extensive analyses conducted for this country-based study. These selected findings are intended to demonstrate the utility of task analysis as a strategic research approach to determination of the scope of practice of health service providers.

Analysis was conducted at the *macro* level, by computation of simple frequencies of performance of various tasks, in any facility or setting. This demonstrated the degree of overall task-sharing, tasks performed by more than one cadre, and tasks performed by cadres outside their expected scope of practice. The 233 tasks were arranged in descending order of priority by cadre, and selected if at least 50% of the respondents reported that they performed the task at the frequency of once per day or once per week (combined). The top 50 tasks are presented in Table 1.

**Table 1:** Frequency of tasks (%) currently performed (per domain/sub domain) by each professional cadre (aggregated data)

**Domains/Sub domains**	**Cadres**					
	**BN**	**GN**	**MCHN-B**	**MCHN- G**	**MA-B**	**MT-M**
**1. Basic health care**						
Basic health actions	**74.7**	**68.5**	**78.7**	**76.2**	**54.3**	**49.2**
1.2. Client hygiene and comfort	**60.5**	**53.6**	**60.6**	**57.8**	28.8	20.6
1.3. Administration of drugs	**70.6**	**63.2**	**69.7**	**67.8**	**43.7**	**21.4**
1.4. Administration of blood	**49.8**	**53.3**	**38.1**	**47.7**	**7.8**	**12.5**
1.5. Inpatient general care	**38.1**	**41.6**	**31.2**	**35.6**	9.4	13.2
1.6. Pre- and post-operative care	**23.5**	**23.4**	**25.9**	**35.6**	3.4	2.3
**2. Obstetrics/gynaecology**						
2.1. Antenatal care	6.0	4.0	**63.8**	**66.9**	18.9	24.4
2.2. Admission - maternity	4.0	1.5	**80.4**	**76.6**	17.3	19.2
2.3. Birth attendance	**4.9**	**2.5**	**72.4**	**66.9**	**15.5**	**12.5**
2.4. Newborn care	4.6	3.0	**75.1**	**69.9**	**15.6**	**12.7**
2.5. Obstetric emergencies	0.9	1.1	**27.5**	**32.4**	5.1	8.6
2.6. Puerperal care up to 6 weeks	3.7	2.0	**57.9**	**62.5**	13.7	12.7
2.7. Family planning and gynaecology	6.1	4.4	**48.0**	**47.1**	18.2	18.8
**3. Child health**	15.4	12.2	**49.5**	**47.0**	**48.7**	**37.6**
**4. General practice**						
4.1. Obtains the clinical history, requests and interprets ancillary tests	21.1	22.5	**27.0**	**33.1**	**49.7**	**78.0**
4.2. Performs the diagnosis	36.3	30.4	**37.6**	**44.2**	**74.5**	**89.8**
4.3. Provides treatment and care	30.2	25.5	**27.1**	**30.6**	**59.0**	**72.0**
**5. Emergency care**	26.7	23.0	12.1	10.3	**26.6**	**33.1**
**6. ART (Antiretroviral Treatment)**						
6.1. Provides counselling and testing	39.9	38.3	**67.3**	**72.0**	**79.9**	**81.4**
6.2. Prescribes adult ART	7.8	12.8	**10.5**	**25.1**	**23.1**	**77.6**
6.3. Prescribes paediatric ART	2.9	5.0	4.6	10.2	10.4	**49.3**
**7. Community outreach**	**6.3**	**5.7**	**10.9**	**9.2**	11.7	11.6
**8. Clinic and programme management**	**31.3**	**47.5**	**32.3**	**44.9**	**36.4**	**38.8**
**9. Training of health personnel**	19.6	**36.0**	17.1	**29.1**	22.8	30.7

The bolded cells indicate that the task is within the scope of practice of the professional cadre(s). BN, basic nurse; GN, general nurse; MCHN-B, maternal/child health nurse – basic; MCHN-G, maternal child health nurse – general, MA-B, medical technician – basic; MT-M, medical technician – mid-level.

**Figure 1:**
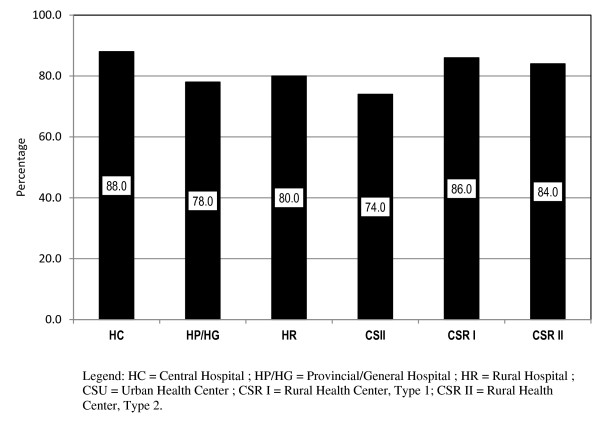
Task sharing of the fifty most frequent tasks among the basic and general nurses by type of health facility.

**Figure 2:**
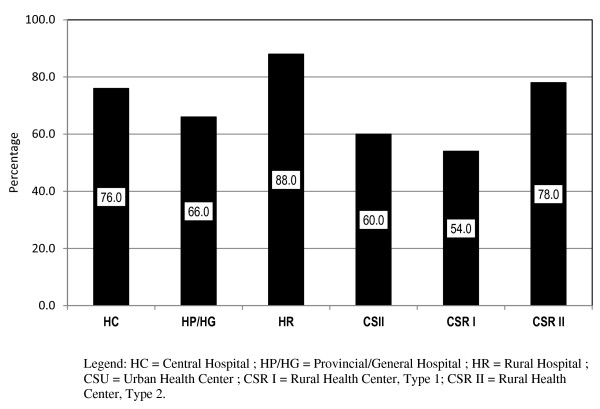
Task sharing of the fifty most frequent tasks among the basic and mid- level MCH nurses by type of health facility.

Analyses were also performed at the *micro* level of analysis, which focused on the tasks that were performed by members of each cadre at the facility level, as it was anticipated that the roles and responsibilities of each cadre would be related to the services that were performed in the various health-service delivery settings. Cross-comparisons were conducted to identify the commonalities in tasks performed by various cadres. Figure 1 depicts task sharing when BNs were compared to GNs; Figure 2 depicts task sharing between MCHN-Bs and MCHN-Gs, within each of the seven types of health facilities.

The tasks performed in common within various facilities by the two cadres of maternal child health nurses indicated a minimum of 75% task sharing within the area of general client care and management, as was the case with BNs and GNs, within central, provincial and rural hospitals. Clear distinctions in scope of practice between the two nurse and two MCHN cadres (B and G) were demonstrated by data indicating that MCHN-B and MCHN-G personnel, rather than basic and general nurses, were responsible for HIV prevention activities and for the obstetric care of women.

Normal delivery services were, in general, provided by both basic and mid-level MCHNs far more frequently when compared to the two levels of nurses and medical technicians, an expected finding, based on the scope of practice. However, in rural health centres type 2 (RHC-II) (the country’s most basic health-service delivery setting), all cadres were called upon to conduct both normal and emergency obstetric care (EmOC) functions in these settings. For example, almost 40% of both medical technicians and medical agents assisted at childbirth at least once a day or once a week in RHC-IIs. The EmOC function of manual removal of the placenta, while never performed by cadres other than MCHNs in higher-level facilities, was in fact conducted at least once per day or once per week by one in every five MTs or MAs, and slightly more than one in every ten nurses, in RHC-IIs.

### Additional functions

A simple aggregate count was made of the tasks that respondents indicated were part of their job responsibilities but for which they had not been prepared in the education programme. Administrative, management and teaching functions were most often cited. Additionally, respondents indicated responsibilities to attend to client triage, administration of medications, provision of emergency-care services (trauma, minor surgery) and emergency obstetric care.

## Discussion

The intention of this study was fundamentally to identify whether there was a need to revise the curricula of study for the various categories of nurses, MCHNs, and MAs/MTs, so that graduates were prepared to perform each of the tasks that were expected of them, in whatever health facility they were assigned. We conducted a full assessment of the content included in the curricula of study for all six cadres, and a field study of tasks actually performed in the workplace, to identify those items of knowledge and skill that were the (then current) expected outcomes of pre-service education for the two pathways of study for the six cadres of health workers, so that it was possible to know whether any cadre was providing services outside of the scope of practice.

The present article focuses only on the findings from the field study (the skills survey) that documented the frequency of tasks actually performed by any of the six cadres in any of the types of health facilities to which they were assigned. We also limit the focus of this article primarily to a discussion of findings related to the nursing careers.

For the two cadres of nurses we identified a minimum of 74% sharing among the most frequently performed (top 50) tasks when task performance was reviewed at specific facility levels. In other words, at least three of every four tasks were performed by the basic and general nursing cadres without distinction on the basis of educational preparation. The tasks most commonly performed in the central, provincial and general hospitals were largely related to the domains of basic health care, administration and management. However, at the health centre levels, nurses performed tasks related to general practice (including diagnosis and treatment that are in line with the scope of practice of medical agents and technicians, rather than of nurses). A consolidation of the education pathway through a standardization of the curriculum of study was determined to be an appropriate strategy for the nursing career, and in line with lessons learned about strategies that have been proven effective for scale up [30].

These findings about task sharing were also identified between the two cadres of MCHNs when knowledge (endorsement of importance) and skills (frequency of performance) data were compared. Task sharing also occurred between MCHNs when compared to the two cadres of nurses (BN and GN). There was substantial, and in some areas, complete task-sharing between the two levels of MCHNs in all general client-care, reproductive, maternal, and infant health domains, indicating, as would be expected, that the content area is held in common, in spite of the differences in length of the education programme. MCHNs were intended to have a distinct scope of practice, for which the expected outcomes of education differed substantially from that of the basic and general nurse, but, nevertheless, they actually practiced many tasks in common.

MCHN cadres shared a common body of knowledge and set of skills, so that it was difficult to distinguish between the responsibilities of graduates from either of the two educational levels within the reproductive health, maternal, newborn and child health domains. Both cadres performed essential basic and emergency obstetric-care functions at approximately equal frequency across all types of health facilities [31]. Fauveau and colleagues [29] note that any advances toward achievement of MDG maternal/child health goals are likely only when country-based maternal health programmes focus on scaling up larger numbers of truly skilled birth attendants, rather than focusing on the production of workers with shorter training and fewer skills. The commonalities in rating of the importance of knowledge areas relevant to the scope of MCH practice and the overlap of tasks that each MCHN cadre provides in the work setting indicated that the two cadres of MCHN personnel should be consolidated into a single educational pathway, through a higher educational career pathway.

There was some task sharing between MCHNs, other nurses, and the two cadres of MAs and MTs, in areas of practice that relate to common client-care, infection prevention and selected primary-care functions, including HIV counselling and treatment [32-34]. MCH nurses at both levels assumed some responsibilities for client triage and emergency-care services for which they were not prepared in their curricula of study. This was clearly demonstrated for MCH nurses who were working in rural health facilities. These findings are echoed by Kruk *et al*. [35] in their cross-national analysis of low- and middle-income countries which demonstrated that a broad mix of health workers accounted for provision of a substantial proportion of health services.

The intention of these analyses were to answer the question, “*What tasks are performed by any one of the cadres, in any setting to which the individual is assigned to work?”,* and to identify similarities and differences in task expectations between and among cadres. Study findings confirmed that there were clearly two cadres of nursing personnel in the country, those who performed tasks in general medical-surgical domains, and those who performed reproductive health care-related functions, that is, a nurse, and an MCH nurse. There was clear evidence of task sharing between the tasks performed by the two levels of nurses (comparing BN to GN) and the two levels of MCH nurses (comparing basic to general), despite differences in their educational preparation for practice in rural health settings, in response to presenting client needs. There was far less distinction when the two levels of BN and GN, and when the two levels of MCH nurses were compared one to the other. There was also clear evidence of task shifting from maternal/child health nurses to GNs, particularly for tasks within the maternity-care domain, likely because of exigent circumstances and facility needs [36-40].

### Limitations

The findings in this report are based on results obtained from a very robust sample of respondents, well-balanced in terms of age and years of experience. Each of the Provinces and Maputo City were well-represented. The full variety of health-care delivery settings, and both in-patient and out-patient service delivery units were included in the sample. The RHC-IIs, were particularly well-reflected. Nevertheless, it cannot be said with certainty that the respondents are proportionately representative of the actual providers of service in each health facility and care setting.

## Conclusions

Overall findings of this study of six provider cadres indicated that in general there was a reasonable match between the scope of practice defined for the professional career and the tasks actually carried out in the health facility. This segmentation of competencies between the professional cadres presents some clear advantages, such as allowing a distinct focus on pre-service education for the specific cadre. However, the disadvantage is that there is a need to plan for facility staffing on the basis of complementary competencies of the provider cadres selected for service in any particular setting.

Study findings indicated that some cadres were underperforming in their scope of work, due in part to their assignment to facilities where the skill mix between cadres was not well-balanced, while in other settings individuals were performing tasks outside of their scope of professional practice for this same reason. This imbalance can lead to “de-skilling”, particularly for clinical conditions that occur at low frequency. It also presents a challenge to the delivery of quality health-care services, when individuals are not appropriately prepared to provide the specific services, or when they are assigned to a practice setting where the work environment does not enable best practice [41]. Study findings indicated a clear need for adherence to training and deployment policies that produce sufficient numbers of appropriately qualified personnel who can be assigned to designated levels of a health facility, so that no health worker is placed in a setting where the provider, often by necessity, finds it necessary to function outside the defined scope of practice.

Findings specific to the nursing cadres indicated that there is a common core of knowledge that would lend itself well to co-teaching across disciplines. There was also a distinct commonality of clinical skills between the two cadres of nurses and between the two cadres of MCHNs. Therefore, it was deemed appropriate to craft a single educational pathway for GNs, as well as a single pathway for maternal/child health nurses.

This newly crafted 24-month curriculum was implemented in February 2011. A total of 453 nurses (163 GNs and 290 MCHNs) have completed studies under these new curricula to date. All students passed the exit examinations, and were deemed eligible for deployment. An assessment comparing quality of competencies of these careers with the former and revised curricula is underway. Results will be available in early 2014.

### Conclusion

The authors believe that the methodology of task analysis can be recommended to other country policy makers and educators as a disciplined and rigorous approach to assessment of the capacity and sufficiency of health-care human-resource personnel. The methodology has a high degree of validity, is highly replicable, and offers substantial utility in its application.

## Abbreviations

BN: basic nurse; EmOC: emergency obstetric care; GN: general nurse; IRR: inter-rater reliability; MA: medical agent; MA-B: basic level medical agent; MCHN-B: basic level maternal/child health nurse; MCHN-G: mid-level maternal/child health nurse; MDG: Millennium Development Goals; MOH: Ministry of Health; MT: medical technician; MT-M: mid-level medical technician; NPHHRD: National Plan for Health Human Resources Development; RHC-II: rural health centre type 2; TA: task analysis; TB: tuberculosis; WHO: World Health Organization.

## Competing interests

The authors declare that they have no competing interests.

## Authors’ contributions

MD, AM, EN, DB, MR, and JF were involved in all aspects of study design, data collection, data interpretation and report writing. All authors participated in critical revision of the manuscript, and all approved the final version.
